# GluK2 kainate receptor subunit-selective, potentiating RNA aptamer

**DOI:** 10.1038/s41598-025-15323-y

**Published:** 2025-09-11

**Authors:** Samantha R. Ingenito, Noah Saunders, Kyle J. Lininger, Li Niu

**Affiliations:** Department of Chemistry, Center for Neuroscience Research and RNA Institute, University at Albany, State University of New York, Albany, NY 12222 USA

**Keywords:** Ion channels, Neurochemistry, RNA, Biochemistry, Molecular biology, Neuroscience

## Abstract

Kainate receptors are a subtype of ionotropic glutamate receptors. Potent and selective modulatory agents of kainate receptors are potential drug candidates for treatment of various neurological diseases involving receptor dysregulation. Here, we report an RNA aptamer that selectively potentiates GluK2, a key kainate receptor subunit. The potentiating aptamer, termed U9, is designed by changing the sequence of the predecessor RNA, which is an inhibitor without subunit selectivity, leading to the change of function: U9 selectively potentiates GluK2 with an EC_50_ of ~ 210 nM. Our study demonstrates a potential utility of combining molecular docking with functional assay for a template-based discovery of potent, single-subunit selective RNA aptamers.

## Introduction

Ionotropic glutamate receptors (iGluRs) mediate the majority of excitatory neurotransmission in the mammalian central nervous system (CNS), and they are essential for brain development and function^1,2^. iGluRs are divided into three subtypes, which include kainate receptors, along with *N*-methyl-D-aspartate (NMDA) and α-amino-3-hydroxy-5-methyl-4-isoxazole propionate (AMPA) receptors^3^. Kainate receptors have five subunits, GluK1-5. GluK1-3 can each form functional channels with tetrameric assembly, whereas GluK4 and GluK5 must each assemble with one of the GluK1-3 subunits to form functional, heteromeric channels^4–10^.

The role of kainate receptors in the CNS function and CNS diseases is the least understood among the three iGluR subtypes^11,12^. This is mainly attributed to a lack of pharmacological tool molecules selectively targeting kainate receptors or individual kainate receptor subunits^13^. To date, virtually all allosteric modulators of kainate receptors are synthetically made^13–15^. Almost all existing compounds are selective to the GluK1 subunit over any other kainate receptor subunits, and many of these compounds have cross activity on AMPA receptors^13,16^. No allosteric modulator, positive or negative, selects the GluK2 subunit, despite the fact that GluK2 is one of the key kainate receptor subunits in the brain^17^. Positive allosteric modulators are also termed as potentiators, since they enhance the activity of a receptor in the presence of an agonist. Potentiators of NMDA and AMPA receptors have been developed, and use of these potentiators has shown therapeutic potential for treating conditions associated with cognitive impairments and/or psychiatric disorders that involve hypoactivity of these receptors^18–21^. However, little is known about positive modulation of kainate receptors in vivo. Concanavalin A (ConA) and other plant lectins are potentiators of kainate receptors, including GluK2, but ConA also potentiates AMPA receptors^22,23^. Several compounds with the core structure of benzothiadiazine, such as BPAM344, do potentiate kainate receptors^24^. However, BPAM344, for example, potentiates both kainate^24^ and AMPA receptors^25^.

We have previously reported an RNA aptamer with dual inhibitory activity for both kainate and AMPA receptors^26^. This aptamer was discovered from the use of systematic evolution of ligands by exponential enrichment (SELEX) with an RNA library containing ~ 10^14^ random sequences. We further separated the two segments of this RNA sequence and created two new aptamers: one inhibits kainate receptors (AB9s-b) and the other inhibits AMPA receptors. However, AB9s-b is not GluK2 subunit selective, because it inhibits both GluK1 and GluK2^26^. Nonetheless, these earlier experiments have suggested that it is possible to use AB9s-b as the structural template to design new RNA aptamers with improved subunit selectivity. Here we describe the success of developing a GluK2 subunit selective RNA aptamer based on the AB9s-b RNA template.

## Results

### Template-based design of AB9s-b sequence variants

**Fig. 1:**
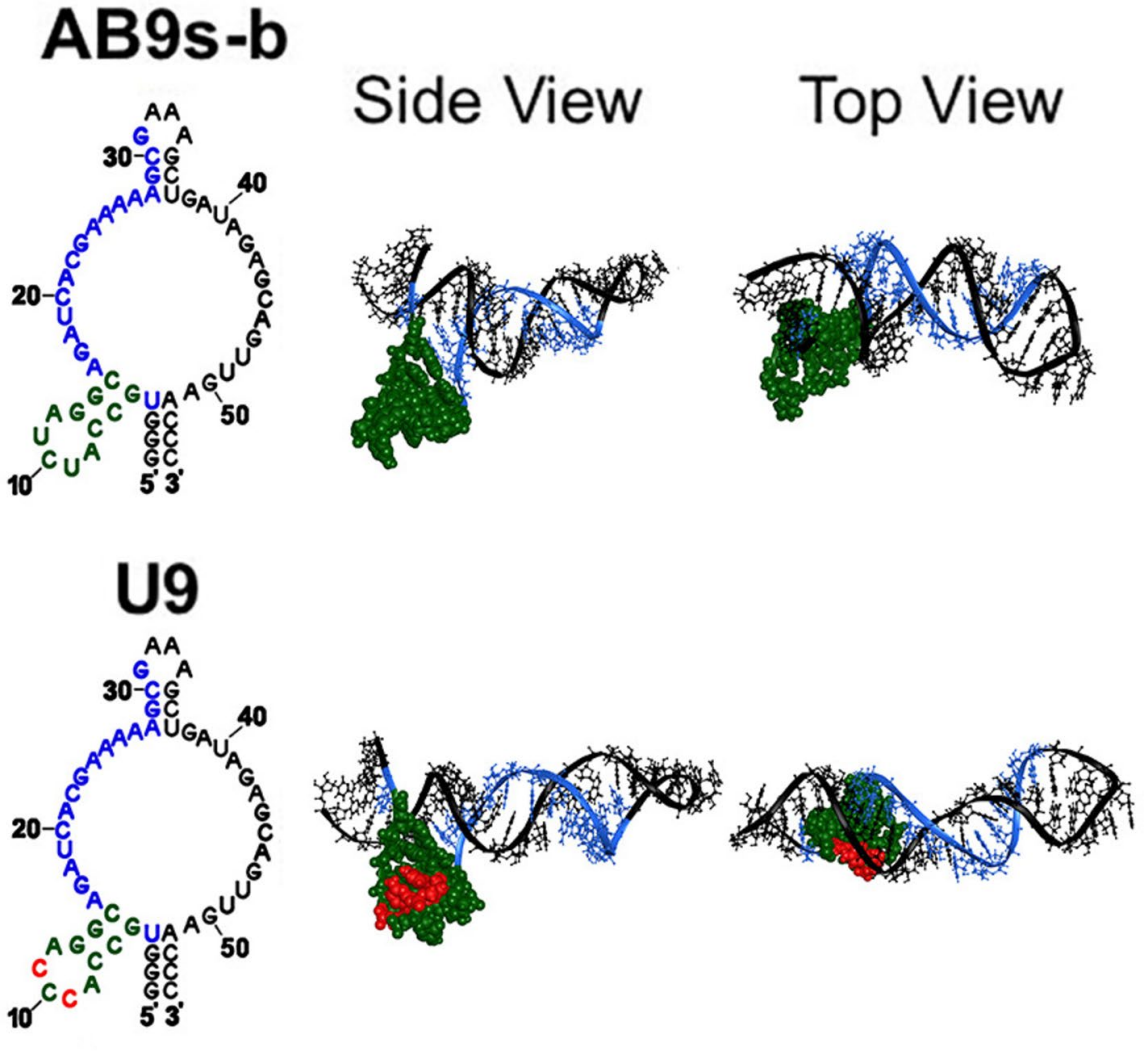
Predicted secondary and tertiary structures of AB9s-b and U9. The upper row shows the secondary structure of AB9s-b (left) predicted by MFold. The blue colored sequence corresponds to the essential sequence segment of the AB9s-b RNA aptamer that inhibits GluK1 and GluK2. The three-base mutation (nt 9–11) is highlighted in red, the pentaloop is shown in green, and the rest of the sequence is in gray color. On the right, the tertiary structure of AB9s-b, generated from FARFAR2 procedure, is shown by a side view and top-down view. Similarly, the lower row displays the secondary and tertiary structures of U9 aptamer whose sequence differs from AB9s-b in the pentaloop, as highlighted in red.

To find a GluK2-selective aptamer, we started with AB9s-b as the template. AB9s-b is a 55-nucleotide (nt) long RNA aptamer originated from the SELEX experiment^26^. AB9s-b inhibits both GluK1 and GluK2 homomeric kainate channels equally potently but without activity on either AMPA or NMDA receptors^26^. Therefore, our goal was to use AB9s-b to create a new RNA aptamer selective to the GluK2 kainate receptor subunit. In other words, the new aptamer would lose the activity on GluK1 and remain inactive in other iGluR subunits. To achieve this goal, we hypothesized that changing the nucleotide sequence of AB9s-b would lead to changing the structure and therefore the function of the resulting RNAs. To test this hypothesis, we selected the pentaloop from nucleotides 9–11 for sequence change (Fig. 1). Pentaloops are known to play various functional roles in RNA, including serving a critical function in group II intron catalytic activity^27^, mediating a wide range of intra- and intermolecular interactions in group I intron^28^, facilitating interactions with RNA helices^29,30^, stabilizing the overall tertiary fold of RNAs^31^ and serving as the site of interaction with various RNA binding proteins^32–36^. Therefore, the fact that pentaloops play important roles in RNA functions, as described above, suggested that changing the sequence of the pentaloop in AB9s-b could change the structure and then the function of a resulting RNA. Based on these design principles, U9 RNA was generated; and U9 was predicted to have a 3D structure different from AB9s-b (Fig. 1).

### Functional characterization of U9

**Fig. 2:**
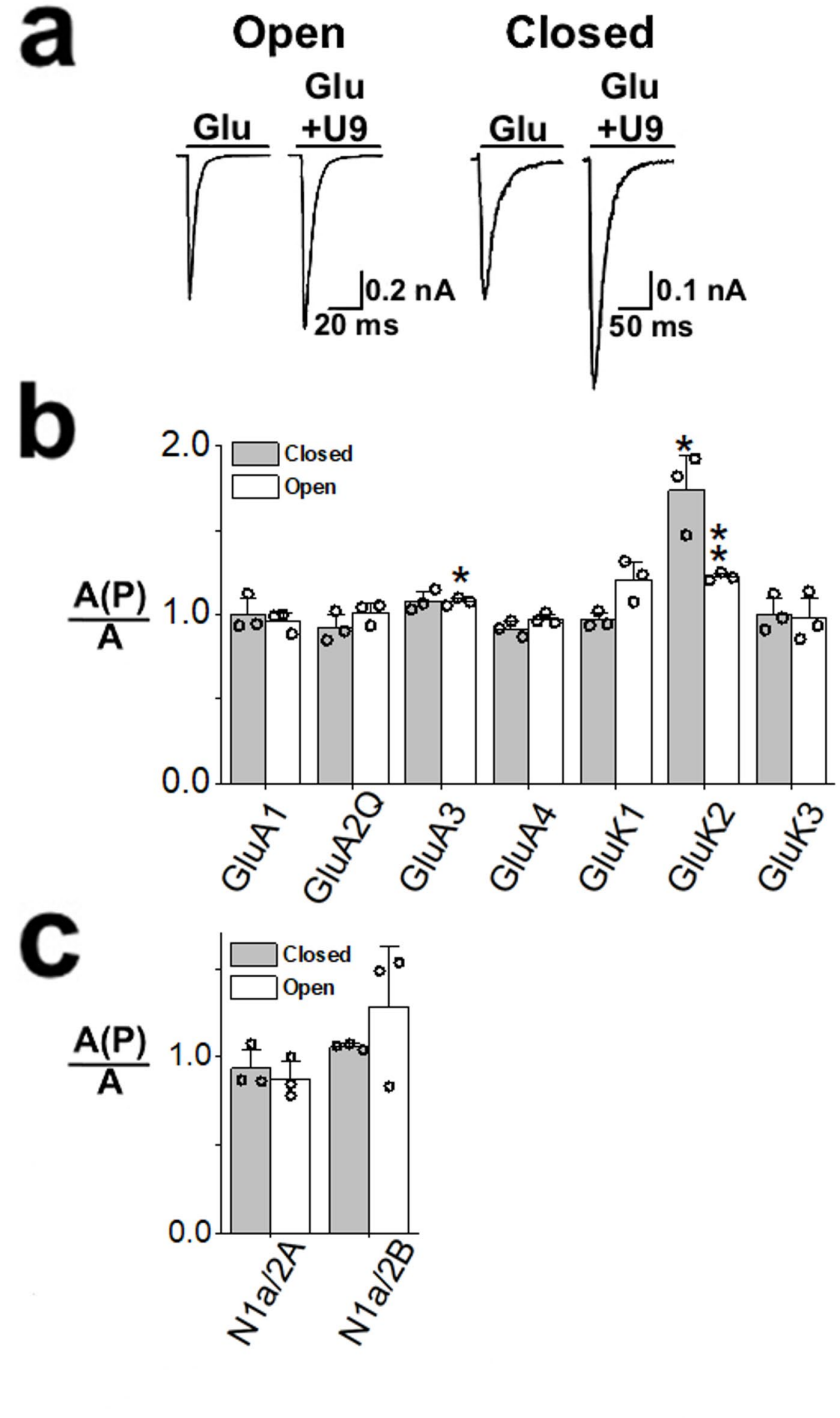
Selective potentiation of GluK2 by U9 aptamer. *A,* Representative whole-cell current responses of GluK2 to 3 mM glutamate (left) and 0.05 mM glutamate (right) in the absence or presence of 2 µM U9. The current amplitude was obtained from whole-cell recording with HEK-293 cells expressing GluK2. *B,* The ratio of whole-cell current amplitude in the presence of 2 μM U9, *A(P)*, and the absence of the aptamer, *A*. The glutamate concentration was chosen to be equivalent to ∼4% and ∼96% fraction of the open channels for each of the receptor subunits and types. Specifically, the glutamate concentration was 0.1 mM for the closed-channel form (solid column) and 3 mM for the open-channel form (hollow column) for GluA1, GluA2Q, GluA3, and GluA4. GluK1 and GluK2 were tested at 0.05 mM glutamate for closed-channel form and 3 mM for the open-channel form. GluK3 was tested at 1 mM glutamate for closed-channel and 20 mM for the open-channel form. The significance in the level of potentiation was determined with a one-sample, two-tailed Student’s t test (*p < 0.05, **p < 0.01); *A(P)/A* = 1 when there is no effect (i.e., neither potentiation nor inhibition). All results are based on at least three measurements and error bars represent the standard deviation from the mean. Comparisons between AMPA receptor and kainate receptor *A(P)/A* values for the closed- and the open-channel forms were analyzed using one-way ANOVA with post-hoc Tukey HSD test analysis, resulting in p values of 0.00962 and 0.000005 respectively. *C*, Selectivity assay of NMDA receptors using 2 µM U9. The glutamate concentration was 0.02 mM and 0.05 mM for both N1a/2A and N1a/2B.

Using whole-cell recording with HEK-293 cells that expressed GluK2 homomeric channels, we assayed the function of U9. As seen by the representative whole-cell current responses to glutamate (Fig. 2), U9 potentiated the GluK2 kainate receptor, unlike its predecessor aptamer AB9s-b, which is an inhibitor of GluK2^26^. Specifically, U9 potentiated GluK2 homomeric channels at low glutamate concentration but had no effect on the GluK2 receptor response at a saturating glutamate concentration (Fig. 2a). We attributed this apparent activity of U9 to its potentiating the closed-channel state, rather than the open-channel state (see Methods). Furthermore, U9 did not have any effect on either GluK1 or GluK3 homomeric kainate channels, nor did it have effect on homomeric AMPA receptors or NMDA receptors that we tested in this study at either low or high glutamate concentrations (Figs. 2b and 2c; supplementary Fig. 1). Even at 6 µM concentration under which the maximum potentiation of GluK2 homomeric channels was achieved (see below), U9 did not have any effect on other homomeric kainate receptors or an AMPA receptor (supplementary Fig. 2).

**Fig. 3:**
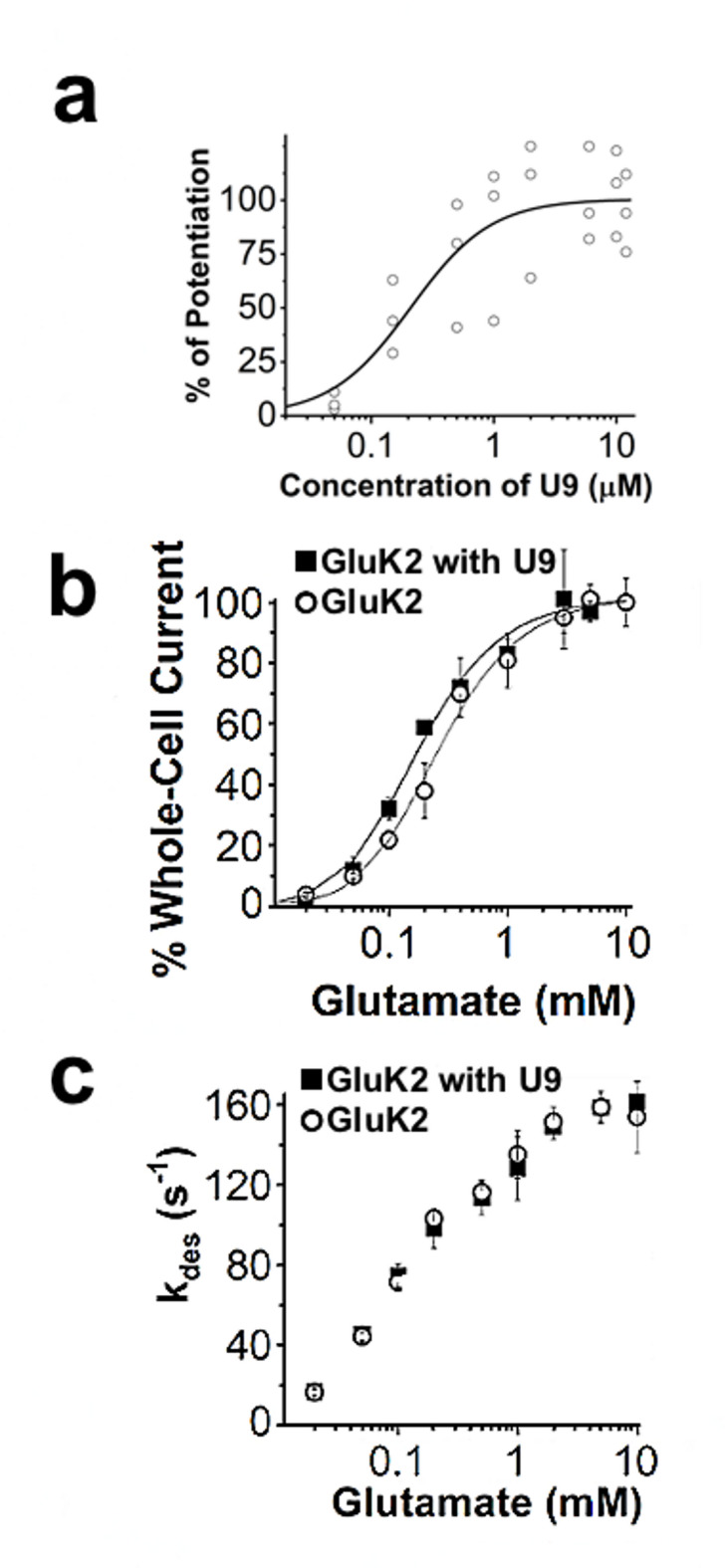
Functional characterization of GluK2 with U9. *a,* The whole-cell current response to 0.05 mM glutamate as a function of U9 concentration. Each symbol represents a single data point. The maximum current response was the average of the responses from U9 concentrations of 6, 10 and 12 µM, and was set to be 100%. The analysis of the dose response by nonlinear regression using the Hill equation yielded EC_50_ of 210 ± 70 nM, and Hill coefficient of 1.3 (the adjusted R^2^ = 0.68). *b,* The dependance of channel desensitization rate constant on glutamate concentration in the absence (o) and presence (■) of 2 µM U9 was determined from the whole-cell current of GluK2. Each point is an average of at least three measurements from three cells. The standard error of the mean is shown. *c,* Dose–response relationship for GluK2 in the absence (o) and presence (■) of 2 µM U9. The average of the current amplitudes at 5 and 10 mM was set to 100% for the dose–response relationship. The dose–response relationship for GluK2 (o) and GluK2 plus U9 (■) was analyzed by nonlinear regression using the Hill equation. The best-fitted parameters from nonlinear regression yielded EC_50_ of 0.26 ± 0.02 mM and 0.18 ± 0.02 mM for the absence and presence of U9, respectively.

We further characterized the effect of U9 on GluK2 homomeric channels. First, we measured the potentiation of GluK2 whole-cell current response as a function of the concentration of U9 but only at low glutamate concentration (Fig. 3a). We found that 2 µM of U9 was high enough to generate the maximum potentiation of GluK2 channels (Fig. 3a). Furthermore, the EC_50_ value of the U9 potentiation was estimated to be 210 ± 70 nM (Fig. 3a). Second, we also characterized the dose–response relationship by varying glutamate concentrations in the presence and absence of U9. As shown (Fig. 3b), U9 caused a left shift of the dose–response curve, indicating that U9 potentiated the GluK2 homomeric channels by making the channel more sensitive to glutamate in that a lower glutamate concentration, such as 0.1 mM, induced a higher percentage of the channels to open when U9 was bound to the receptor. The fact that the dose–response curves with and without U9 converged at saturating glutamate concentrations further suggested that U9 had no significant effect on the open-channel conformation, again, consistent with the results in Figs. 2a and 2b that U9 would be bound to an allosteric site on GluK2. Lastly, from every glutamate concentration we tested, our results showed that U9 potentiated the current response but without any effect on the rate of channel desensitization or k_des_ (Fig. 3c).

### Molecular docking study of U9 with the GluK2 receptor

The change of the nucleotide sequence in the stem-pentaloop in the AB9s-b aptamer clearly resulted in the improvement of the selectivity of the new U9 RNA aptamer. This was evident that U9 selected GluK2 without any effect on GluK1, unlike AB9s-b. However, the change in function from AB9s-b as an inhibitor on GluK1 and GluK2 to U9 as a potentiator on GluK2 alone was unexpected, especially since only two bases were different between the two aptamers. To better understand the origin of this potentiation by U9, we conducted molecular docking study of U9 with several relevant receptors.

**Fig. 4:**
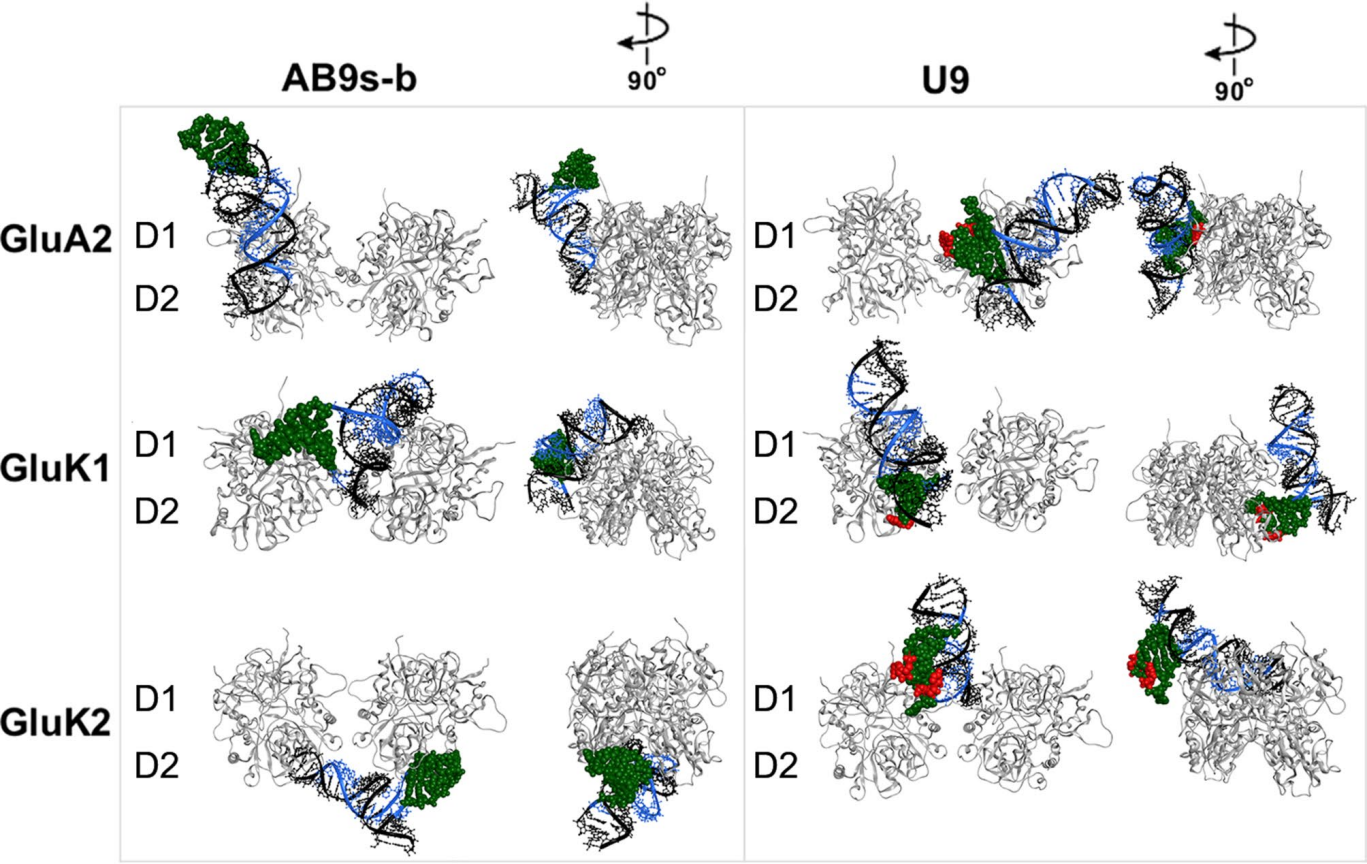
Predicted docking modes of AB9s-b and U9 to GluK2, along with GluK1 and GluA2. ZDock predictions of docking of AB9s-b and U9 to GluK1, GluK2, and GluA2, as the most stable conformation according to the DRPscore method. Only the LBD of each receptor in the closed-channel conformation is shown for the front-facing view and a 90° rotation to the side view. The site of the mutated hairpin (nt 5–15) is highlighted in red.

First, the most stable tertiary structures of AB9s-b and U9 were generated, based on secondary structure predictions from MFold, using Fragment Assembly of RNA with Full-Atom Refinement 2 (FARFAR2) scoring^37^. As shown in Fig. 1, the two-base mutation (i.e., from UCU in the pentaloop of AB9s-b to CCC in U9) was enough to change the original structure of AB9s-b to a different fold in U9. We then docked these predicted tertiary RNA folds to both GluK1 and GluK2. For a receptor control, we also docked these RNAs on GluA2, an AMPA receptor subunit, since neither AB9s-b nor U9 had any functional effect on GluA2. Shown in Fig. 4 is the most stable structure for each RNA/receptor complex as predicted by DRPScoring, a deep learning-based approach for identifying native-like RNA–protein structures^38^. From this RNA folding/docking experiment, we observed some interesting features. (i) Both AB9s-b and U9 were shown to dock onto the GluA2 receptor roughly in the same area (Fig. 4, upper panel). This prediction was consistent with the result that U9, like its predecessor AB9s-b, had no effect on GluA2 (Fig. 2b). (ii) On the GluK1 kainate receptor, AB9s-b was predicted to bind differently as compared with U9 (Fig. 4, middle panel). This result was consistent with our previous observation that AB9s-b inhibited GluK1 homomeric channels^39^, yet U9 was no longer effective in inhibiting GluK1. (iii) On the GluK2 kainate receptor, AB9s-b was revealed to dock onto the ligand binding domain (LBD) of GluK2, whereas U9 inserted itself at the interface of the two dimers (Fig. 4, lower panel, and supplementary Fig. 3). This predicted docking pose of U9 on GluK2 would suggest that U9 was no longer an inhibitor given that the predicted docking pose of U9 on GluK2 was different from that of AB9s-b. Furthermore, the docking of U9 to the interface of the two dimers on GluK2 would correspond to the effective way of potentiating the GluK2 homomeric channel. The results from these molecular docking predictions indicated that the change of nucleotide sequence from AB9s-b to U9 effectively changed the structure of U9 and then its site of interaction with both GluK1 and GluK2, as compared to AB9s-b. Consequently, the change of the site therefore led to the change of the function for U9, as compared to AB9s-b.

### Making and functionally testing U9 sequence variants, generated by molecular docking prediction

**Fig. 5:**
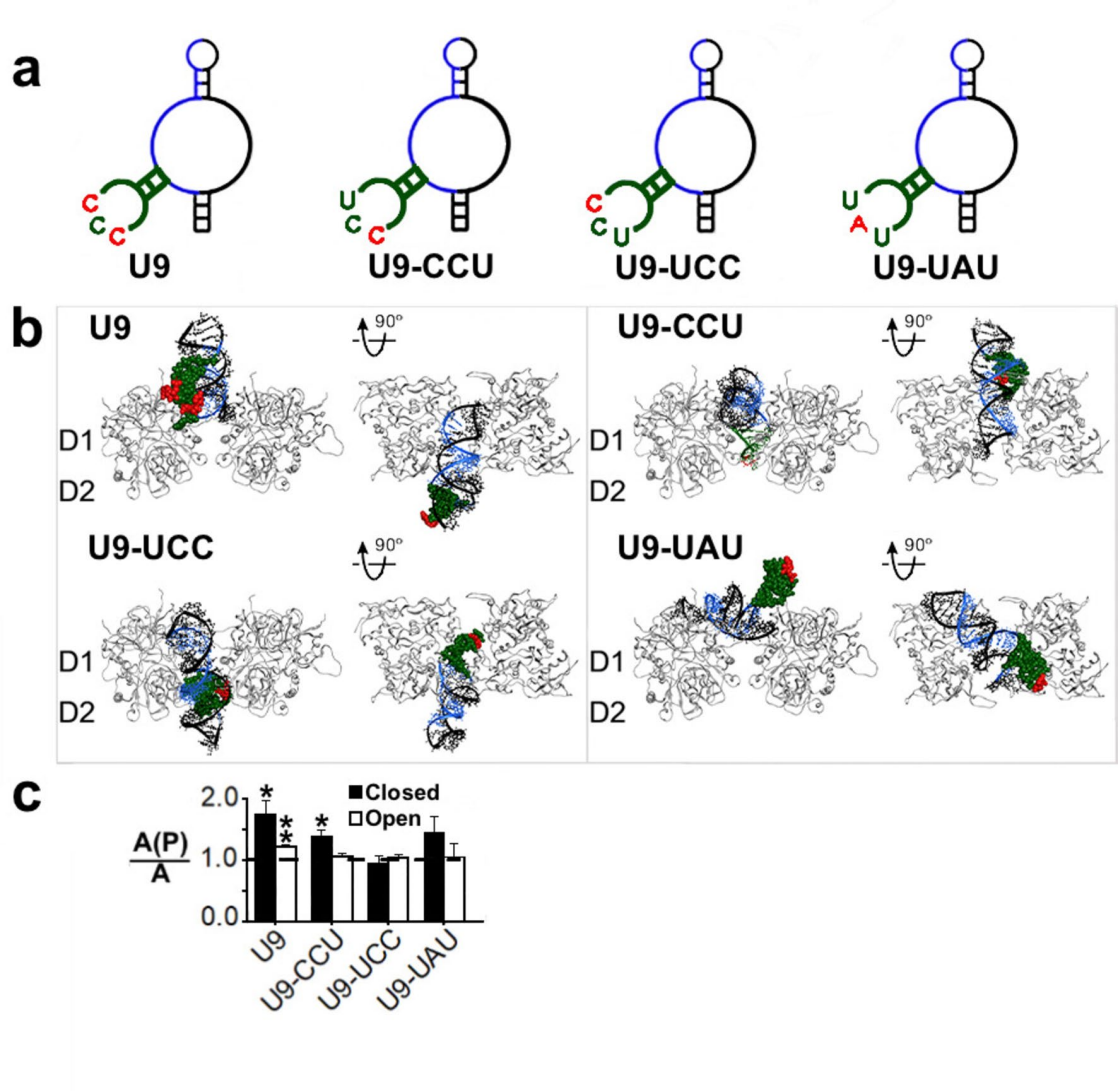
Predicted structures and functional test of U9 sequence variants. *a*, Mfold predictions of the secondary structures of the U9 sequence variants. These variants differ in the sequence in the hairpin highlighted in red. The other color coding is the same as described in Fig. 1 legend. *b,* ZDock predictions of the docking of U9 and its variants on GluK2. Only the LBD of each receptor in the closed-channel conformation is displayed for both the front-facing view and a 90° rotation to the top-down view. The site of the mutated hairpin (nt 5–15) is highlighted in red. *c,* The *A(P)/A* ratio or the ratio of whole-cell current amplitude in the presence of an aptamer (2 μM) and the absence of the aptamer. The glutamate concentration was chosen to be 0.05 mM for closed-channel (solid column) and 3 mM for the open-channel form (hollow column) respectively. The significance in the level of potentiation was determined with a one-sample, two-tailed Student’s t test (*p < 0.05, **p < 0.01); *A(P)/A* = 1 when there is neither potentiation nor inhibition. All results are based on at least three measurements and error bars represent the standard deviation from the mean.

Based on the molecular docking prediction of how U9 was bound to the GluK2 structure, we then hypothesized that an RNA molecule that binds to the interface of the two protomers would potentiate the receptor, like U9. To test this hypothesis, we made additional sequence variants of the U9 RNA. In each of the sequence variants, we made a single nucleotide replacement in the U9 sequence (Fig. 5a, and Supplementary Fig. 4). In fact, some of these sequence changes restored the original nucleotide sequence of AB9s-b (i.e., the sequence of AB9s-b is shown in Fig. 1). For example, the U9-CCU variant differs from U9 whose sequence is CCC, whereas the sequence of AB9s-b is UCU (see their complete sequences in Supplementary Table 1).

The design of these U9 sequence variants was further aided by the molecular docking study of these RNAs with GluK2, the same approach as previously described (Fig. 4b). U9-CCU exhibited a similar fold to U9, and the site where this RNA interacted with the receptor was also similar to the site where U9 made contact with GluK2. As such, U9-CCU was found to be a potentiator, as predicted. U9-UCC, however, was found tilted away from the dimer-dimer interface (Fig. 5b). The lack of interaction with the dimer-dimer interface suggested that this RNA was most likely ineffective in potentiating the receptor. Our functional assay indeed showed that this RNA variant was ineffective on GluK2 (Fig. 5c). The U9-UAU variant seemingly showed little similarity in its pose and also its site of interactions with GluK2, as compared with U9 (Fig. 5, and Supplementary Fig. 4). Yet, a closer examination of this RNA-receptor complex from a different angle (i.e., from top view) showed that this RNA would still bind to the dimer-dimer interface, albeit with a different contact. In other words, U9 contacted the GluK2 receptor mostly along the dimer-dimer interface. In contrast, U9-UAU contacted GluK2 mostly from the top of the dimer-dimer interface. Taken together, these results supported the notion that an RNA predicted to occupy the dimer-dimer interface of GluK2 would be capable of potentiating the receptor activity.

## Discussion

In developing a drug candidate, lack of target selectivity is generally associated with strong side effects. For minimizing or ideally eliminating side effects, a drug candidate should act only on the intended target. Here we described the discovery of U9, a GluK2 subunit-selective RNA aptamer, and U9 does not have any significant activity on any other glutamate ion channel receptors that we tested in this study. We created U9 by using the parent RNA, AB9s-b, as the template. AB9s-b is an inhibitor, and it inhibits both GluK1 and GluK2 homomeric channels roughly equally with an IC_50_ of 3–4 µM^39^. In contrast, U9 is GluK2 subunit-selective, and it potentiates GluK2 homomeric channel with an EC_50_ of ~ 210 nM. As a comparison with existing positive modulators for kainate receptors, neither BPAM344, a small molecule compound, nor ConA, a carbohydrate-binding protein, possesses a kainate receptor subunit selectivity; in fact all of the existing positive modulators of kainate receptors additionally potentiate AMPA receptors^22–25,40^.

The quest for developing a highly selective allosteric modulator for GluK2 has been elusive. Small molecule compounds are generally designed to bind into a cavity on the target in order to affect the target activity. RNA aptamers, like U9, are much bigger, and RNA molecules could bind topographically to the surface of its target with a larger area of contacts so that the interaction could become more selective. A large area of contacts between an RNA and its target could also take advantage of potentially favorable surface properties of the target, such as hydrogen bonding and Van der Waals interactions^41–43^. Although we do not know the precise molecular detail of how U9 interacts with the GluK2 receptor from the docking prediction, we speculate that extensive, unique interactions between U9 and the GluK2 kainate receptor, and a lack of interactions with other glutamate receptor subunits, are responsible for U9 aptamer’s subunit selectivity.

The success of this work in generating the U9 aptamer (and additionally its sequence variants, U9-CCU and U9-UAU) has validated our choosing the pentaloop for making sequence changes to generate new RNA aptamers for higher selectivity. We also attempted to make sequence changes at minimal nucleotide positions, even within the pentaloop, so that we would not lose the selectivity of AB9s-b (or its kainate receptor subtype selectivity) in any mutant RNAs. The fact that U9 sequence variants with single-base changes, which are also single-base sequence variants of AB9s-b (Figs. 1 and 5), are functional shows that each of these nucleotide positions, i.e., U9, C10 and U11, is critical in determining both the folding and the function of the RNA. For example, replacing C with U at position 9 on U9, i.e., U9-UCC, results in losing the potentiating activity of U9. This replacement is also equivalent to replacing U with C at position 11 on AB9s-b, which results in losing the inhibitory property of AB9s-b. Additional sequence changes at C10 and U11, together with the “wildtype” sequence of AB9s-b, and the resulting changes of their functions also served as controls to each other to demonstrate the critical positions and the nature of the sequences at these positions in defining the properties of these RNA variants. All of these results further suggest a potential utility of making additional base changes or even loop size changes to generate more RNA aptamers with newer modulatory properties.

The molecular docking experiment revealed that U9 potentiation of the GluK2 receptor is most likely through binding to the interface of the two dimers. Dimer movement is considered a crucial structural change in the glutamate receptor gating process^44^. U9 inserts itself into the dimer-dimer interface, which may stabilize the interface due to its extensive molecular contacts to the interface. Stabilizing dimer interface causes the open channel to dwell longer, thereby potentiating the channel. Recent structural studies have revealed that BPAM344 binds to the dimer interface of GluK2^40,45^ as well as GluK1^24^, thereby stabilizing the open channel conformation. Binding of positive allosteric modulators to the dimer interface has been studied more extensively from both crystal structures of isolated AMPA receptor ligand-binding domains and full-length receptors^16,24,44,46–49^. Therefore, it is conceivable that similar RNA aptamers may be also developed to bind to AMPA receptor subunits by poses similar to U9 on GluK2. Whether this is a general design principle of developing potent, selective potentiators for not only kainate but AMPA receptors as well awaits further study. It should be noted that we previously identified an aptamer that selectively inhibits the GluA2 AMPA receptor subunit using SELEX with a large RNA library^50^. Such a strategy is used to identify RNA aptamers without any pre-defined molecular scaffold for the target. In contrast, the strategy we presented here is faster in producing potentially high performance aptamers, because it is template-based, and the design of sequence variants can be aided by molecular docking predictions.

Positive allosteric modulation could be a preferred therapeutic approach for a target whose activity and/or expression are lower than normal. This is because positive modulators alter the effects of endogenous ligands, and therefore, positive modulators are limited to working in concert with their endogenous ligands on the same spatial and temporal scales. On this note, having the GluK2-subunit selective U9 RNA aptamer should contribute to future studies of GluK2-containing kainate receptors. It is reported that even from early ages of the brain development kainate receptors are widely expressed throughout the nervous system^51,52^. In the adult rat brain, the Grik2 mRNA is most abundant in cerebellar granule layer^53^ (Grik2 is the gene that encodes rodent GluK2 subunit). As evidenced in electrophysical recording, some hippocampal neurons may even express GluK2Q homomeric channels, and these channels are thought to be the unedited in the Q/R (glutamine/arginine) site in transmembrane domain 2 but fully edited in both the I/V (isoleucine/valine) and the Y/C (tyrosine/cysteine) sites in transmembrane domain 1^54,55^. Furthermore, the expression of GluK2 is downregulated in weaver mice or precisely weaver granule cells^56^. In schizophrenia, the mRNA level for the GluK2 subunit is significantly reduced in hippocampus^57^. Therefore, an example of a GluK2 positive modulator like U9 may be useful is to study systems where the GluK2 expression level is abnormally lower. We believe that testing U9 in animals and animal models of diseases with cognitive deficits that involve lower activity of GluK2-containing kainate receptors would provide critical information about whether U9 is therapeutically useful.

## Methods

### RNA aptamer transcription and purification

All of the RNA samples were transcribed enzymatically overnight at 37 °C and then purified by using a cylindrical, 10% urea polyacrylamide gel electrophoresis column, as described^58^. The gel was run in 1 × TBE (Tris borate-EDTA) buffer at 150 V with a flowrate of 1.5 mL/min. The pooled fractions were concentrated using Amicon filtration device with a 10-kDa molecular mass cutoff and buffer-exchanged from 1xTBE to 1 × extracellular buffer (described below). The concentration of the RNA sample was determined using a Nanodrop 1000 spectrophotometer and standardized for each functional assay.

### 3D modeling of RNA aptamers and docking simulations

The tertiary structure of each of the RNA aptamers was first predicted by taking the Mfold secondary structure and inputting it into the FARFAR2 program in which 100 possible RNA tertiary structures were generated and ranked using the FARFAR2 scoring method^37^. Homomeric iGluR models were created by using the resolved heteromeric structures from the RCSB Protein Data Bank (RCSB PDB) and superimposing the sequence of the desired subunit into the position of all the subunits to simulate a homomeric receptor^59–62^. The ligand binding domain (LBD) was isolated from the iGluR models and partially modeled residues were corrected using the molecular operating environment (MOE). Using ZDock, the most stable tertiary structure of an RNA was docked to each iGluR model, creating six hundred possible complexes, which were then ranked by the use of the DRPscore method^38^. The model with the most stable conformation was analyzed through MOE. A distance restraint of 6 Å from the receptor determined whether the nucleotides made a contact or not. Parameters for docking the RNA–protein complexes were based on the AMBER99 forcefield^63^.

### Cell culture and transient receptor expression

The glutamate receptors were transiently expressed in HEK-293S cells with cDNAs encoding the rat GluA1–4 AMPA receptors, GluK1-3 kainate receptors, and NMDA receptors GluN1a/2A and GluN1a/2B that were prepared as previously described^64^. The cells were cultured in Dulbecco’s modified Eagle’s medium (DMEM), supplemented with 10% fetal bovine serum (FBS) in the presence of 100 U/mL penicillin–streptomycin in a 37 °C, 5% CO_2_, humidified incubator. The receptors were co-transfected with large T-antigen and green fluorescent protein (i.e., the green fluorescent protein was expressed as the transfection marker). After 48 h, cells were used for electrophysiology.

### Whole-cell recording

The activity of a putative RNA aptamer was measured by whole-cell recording at − 60 mV and 22 °C, as described previously^65^. The electrode used for whole-cell recording was filled with an intracellular buffer containing 110 mM CsF, 30 mM CsCl, 4 mM NaCl, 0.5 mM CaCl_2_, 5 mM EGTA, and 10 mM HEPES (pH 7.4, adjusted using CsOH). The extracellular buffer contained 150 mM NaCl, 3 mM KCl, 1 mM CaCl_2_, 1 mM MgCl_2_, and 10 mM HEPES (pH 7.4). Both the intracellular and extracellular buffers were adjusted for testing NMDA channels. The intracellular buffer contained 140 mM CsCl, 1 mM MgCl_2_, 0.1 mM EDTA, and 10 mM HEPES (pH 7.2 adjusted by Mg(OH)_2_), and the extracellular solution contained 2 μM of glycine, 135 mM NaCl, 5.4 mM KCl, 1.8 mM CaCl_2_, 10 mM glucose, and 5 mM HEPES (pH 7.2 adjusted by NaOH). Whole-cell recording was performed with an Axopatch-200B amplifier (Axon Instrument) at a cutoff frequency of 2 − 20 kHz by a built-in, 4-pole Bessel filter and digitized at a 5 − 50 kHz sampling frequency by a Digidata 1322 A (Axon Instruments). The whole-cell current response to glutamate was analyzed and reported by *A(P)/A*, the ratio of whole-cell current amplitudes in the presence and absence of a potentiating aptamer^64^.

The green fluorescence in transfected cells was visualized using an Axiovert S100 Carl Zeiss microscope with a fluorescent detection system. Glutamate with and without aptamer was dissolved in external buffer and was applied to an HEK-293 cell in suspension using a rapid solution flow system^66,67^. This system consisted of a U-tube with an aperture of ~ 150 μm. The linear flow rate, controlled by two peristaltic pumps, was 4 cm/second. The rise time generated from 1 mM glutamate for GluK2 was 1 ms^67^. We measured the effect of U9 on various glutamate receptor channels at both low and high glutamate concentrations. Specifically, at a low glutamate concentration (e.g., the one that corresponded to ~ 4% of the concentration that generates the maximum current response), the majority of the receptors in the receptor population would be in the closed-channel state, which included unliganded and liganded forms of the receptors. Therefore, the dominating effect of an RNA aptamer, measured through the whole-cell current amplitude, would be on the closed-channel state of the receptors^26,64,68,69^. Conversely, at a saturating concentration of glutamate, the majority of the receptors in the receptor population would be in the open-channel state. In GluK2, which U9 potentiated, the channel-opening probability of GluK2 channels is 0.96, as we previously determined^67^. By comparison, the channel-opening probability for all other AMPA and GluK1 receptors that we determined ranges between 0.93 and 0.99^66,70–73^. From literature, the peak open probability for GluK3 is 0.5^74^, and the channel opening probability for various NMDA receptors ranges between 0.78 and 0.011^75–79^.

### Statistical data analysis

Each data point used for the *A(P)/A* plot was an average of at least three measurements each of which was from an individual cell. Uncertainties are representative of the standard deviation from the mean. The significance of potentiation was evaluated by a one-sample two-tailed Student’s t test with the null hypothesis being H_0_: μ = μ_0_ = 1, 1 being the theoretical value of no potentiation. An asterisk indicates *p* ≤ 0.05, whereas the two asterisks indicate *p* ≤ 0.01. One-way ANOVA with post-hoc Tukey HSD test analysis was performed to compare the AMPA receptor data with the kainate receptor data or *A(P)/A* values for the closed-channel and open-channel results. The plots were generated using Origin 2020.

## Supplementary Information

Supplementary Information.

## Data Availability

The datasets generated and/or analyzed during the current study are available in the GenBank repository, with accession codes PQ596508 (AB9s-b), PQ596509 (U9), PQ596510 (U9-CCU),PQ596511 (U9-UCC) and PQ596512 (U9-UAU). Correspondence and requests for materials should be addressed to Li Niu.
