# Supplementary material for: GluK2 kainate receptor subunit-selective, potentiating RNA aptamer
Source: Sci Rep. 2025 Sep 11;15:32405. doi: 10.1038/s41598-025-15323-y (PMC12426214; doi:10.1038/s41598-025-15323-y)
Supplement: Supplementary file 1 — Supplementary Information. [file 41598_2025_15323_MOESM1_ESM.pdf]

Supplementary Information for

GluK2 kainate receptor subunit-selective, potentiating RNA aptamer

Samantha R. Ingenito, Noah Saunders, Kyle J. Lininger, and Li Niu*

Table S1

Aptamer Name	*Sequence*
AB9s-b	5′-GGGUGCCAUCUAGGCAGAUCACGAAAAAGCGAAAGCUGAUAGAGCAGUUGAACCC-3′
3sb-U9	5′-GGGUGCCACCCAGGCAGAUCACGAAAAAGCGAAAGCUGAUAGAGCAGUUGAACCC-3′
U9-CCU	5′-GGGUGCCACCUAGGCAGAUCACGAAAAAGCGAAAGCUGAUAGAGCAGUUGAACCC-3′
U9-UCC	5′-GGGUGCCAUCCAGGCAGAUCACGAAAAAGCGAAAGCUGAUAGAGCAGUUGAACCC-3′
U9-UAU	5′-GGGUGCCAUAUAGGCAGAUCACGAAAAAGCGAAAGCUGAUAGAGCAGUUGAACCC-3′

Table S1. Sequences of AB9s-b, U9, and the U9 sequence variants. The three-base mutation area is boxed, while the rest of the sequences are the same for all aptamers.

Figure S1

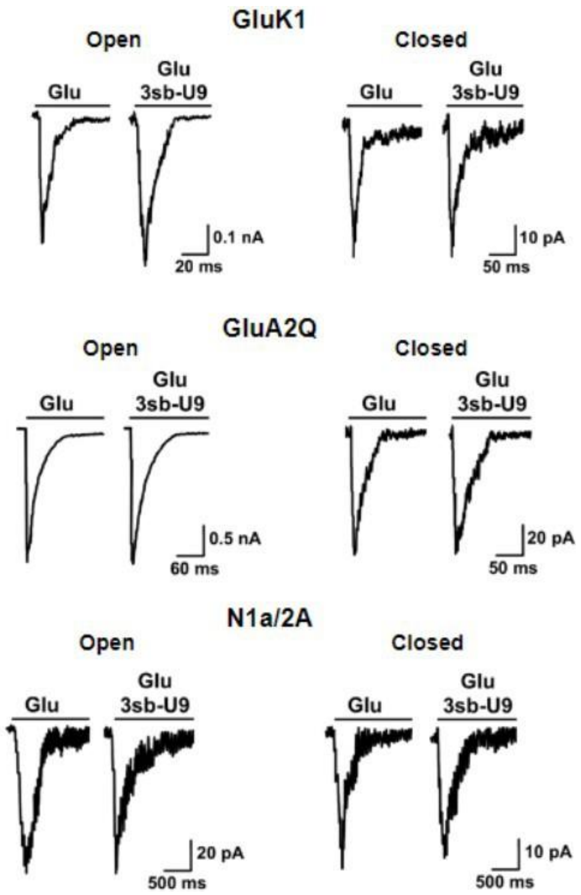

Figure S1. Additional representative whole-cell traces of iGluRs with U9. Representative whole-cell current responses are shown of GluK1 to 3 mM glutamate (top left) and 0.05 mM glutamate (top right), GluA2Q to 3 mM glutamate (middle left) and 0.1 mM glutamate (middle right), and GluN1a/2A to 0.05 mM glutamate (bottom left) and 0.02 mM glutamate (bottom right), in the absence or presence of 2 µM U9. U9 did not have a significant effect on the current responses of GluK1, nor the other iGluRs.

Figure S2

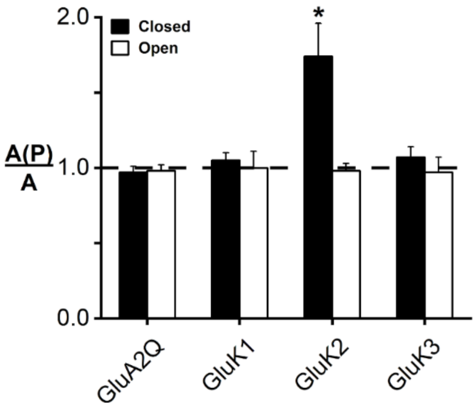

Figure S2. Selectivity assay of U9 at 6 μM concentration with GluK1-3 and GluA2Q. The ratio of whole-cell current amplitude in the presence and absence, *A(P)/A*, of U9. The glutamate concentration was chosen to be equivalent to ~4% and ~96% fraction of the open channels for each of the receptor subunits and types. Specifically, the glutamate concentration was 0.1 mM for the closed-channel form (solid column) and 3 mM for the open-channel form (hollow column) for GluA2Q. GluK1 and GluK2 were tested at 0.05 mM glutamate for closed-channel form and 3 mM for the open-channel form. GluK3 was tested at 1 mM glutamate for closed-channel and 20 mM for the open-channel form. The significance in the level of potentiation was determined with a one-sample, two-tailed Student's t test (*p<0.05, **p<0.01); *A(P)/A*=1 when there is no effect (i.e., neither potentiation nor inhibition). All results are based on at least three measurements and error bars represent the standard deviation from the mean.

Figure S3

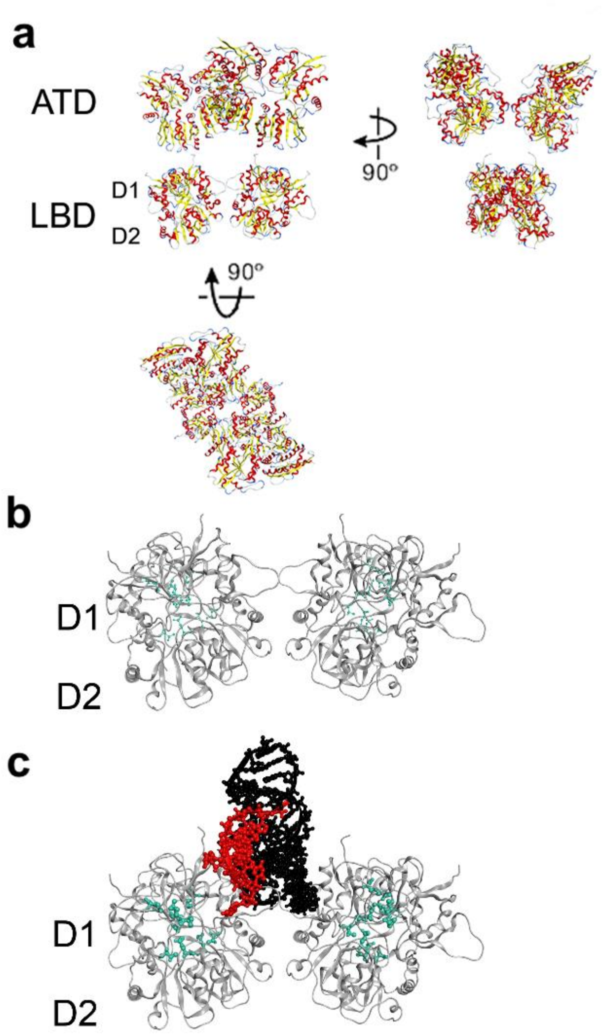

Figure S3. Predicted docking of U9 to GluK2. *a,* The GluK2 model (PDB ID:5KUH) (Meyerson, J., Chittori, S., Merk, A. *et al.* Structural basis of kainate subtype glutamate receptor desensitization. *Nature* **537**, 567–571 (2016). https://doi.org/10.1038/nature19352) was used for molecular docking experiment. The structure is rotated 90 degrees to the side to match the perspective of the side view in Fig 4 and the 90° rotation to the top-down view in Fig 5. The Amino terminal domain (ATD), Ligand binding domain (LBD), the D1 and D2 domain were labeled accordingly. *b,* The GluK2 model (PDB ID:5KUH) with the LBD isolated and colored to match the rest of the structures in the paper. The glutamate binding sites are highlighted in cyan. *c,* ZDock prediction of how U9 binds to GluK2 in comparison to the location of the glutamate binding sites, after finding the most stable conformation according to the DRPscore method. The site of the mutated hairpin (nt 5-15) is highlighted in red. The glutamate binding sites are highlighted in cyan.

Figure S4

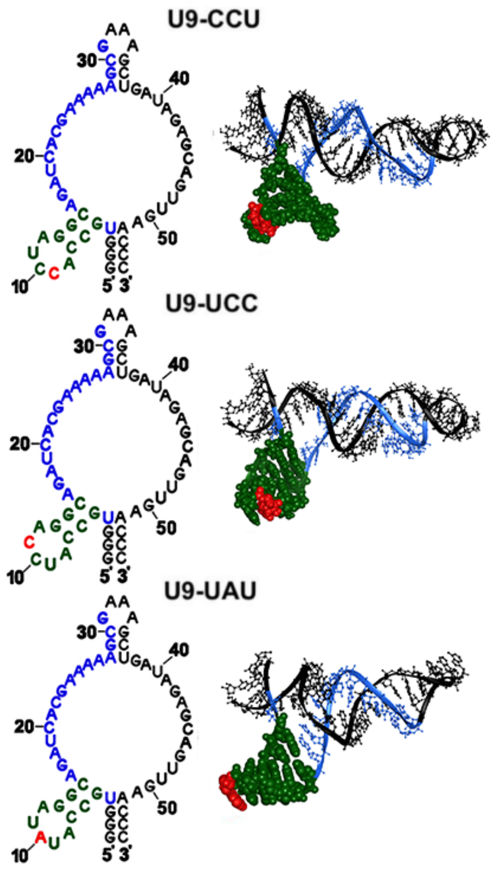

Figure S4. Predicted secondary (left) and tertiary structures (right) of U9 sequence variants. Mfold predictions of the secondary structures of the U9 variants with the sequence that inhibits GluK1 and GluK2 in blue, the mutated section of AB9s-b highlighted in red, and the rest of the conserved sequence in gray. The Mfold structures were then used to predict the tertiary structure of the U9 variants through the FARFAR2 scoring method. The site of the mutated hairpin (nt 5-15) is highlighted in green with a sphere representation for the atoms. Mutated nucleotides from the AB9s-b sequence are highlighted in red, and the blue region from AB9s-b is highlighted in blue.
